# Sphingolipid metabolism-related genes *B4GALNT1* and *CERS4* as prognostic biomarkers in lung adenocarcinoma

**DOI:** 10.1371/journal.pone.0340437

**Published:** 2026-02-10

**Authors:** Jieun Jeon, Junho Kang, Jae-Hyung Park, Jae-Ho Lee, Dong Eun Kim, Woo-Jae Park, Shin Kim

**Affiliations:** 1 Department of Immunology, School of Medicine, Keimyung University, Daegu, Republic of Korea; 2 Department of Research, Keimyung University Dongsan Medical Center, Daegu, Republic of Korea; 3 Department of Physiology, School of Medicine, Keimyung University, Daegu, Republic of Korea; 4 Department of Anatomy, School of Medicine, Keimyung University, Daegu, Republic of Korea; 5 Department of Biochemistry, Chung-Ang University College of Medicine, Seoul, Republic of Korea; 6 Institute of medical science, School of Medicine, Keimyung University, Daegu, Republic of Korea; 7 Institute for Cancer Research, School of Medicine, Keimyung University, Daegu, Republic of Korea; University of Pisa Department of Biology: Universita degli Studi di Pisa Dipartimento di Biologia, ITALY

## Abstract

Sphingolipid metabolism is an important component of various biological processes, particularly in cancer pathology. This metabolic pathway significantly influences the behavior of cancer cells by regulating growth, apoptosis, and survival. Although modulating sphingolipid metabolism has attracted attention as a novel therapeutic strategy, its complexity and specific mechanisms remain incompletely understood. In the current study, transcriptomic profiling was employed to compare the expression of sphingolipid metabolism-related genes between normal solid tissues and lung adenocarcinoma tissues. Additionally, Gene Ontology, Kyoto Encyclopedia of Genes and Genomes enrichment, and protein–protein interaction (PPI) analyses were performed to investigate the functional relevance of these genes. Twelve sphingolipid metabolism-related genes formed a highly interconnected core, suggesting their potential central role within the regulatory network. Four genes were found to be significantly correlated with overall survival. Notably, *B4GALNT1* upregulation and *CERS4* downregulation correlated with advanced tumor stage and metastasis. They also showed prognostic significance in Cox regression analyses, and these findings were consistently validated in an independent cohort. *In vitro,* within lung adenocarcinoma cell lines, *B4GALNT1* knockdown and *CERS4* overexpression suppressed cell proliferation, migration, and epithelial-to-mesenchymal transition, supporting their roles in lung adenocarcinoma progression. These findings highlight *B4GALNT1* and *CERS4* as potential prognostic biomarkers and therapeutic targets in lung adenocarcinoma, warranting further clinical investigation.

## Introduction

Lung adenocarcinoma (LUAD) has a poor prognosis, posing significant challenges in developing effective therapeutics [1,2]. Although the survival rate of early-diagnosed LUAD has improved due to advancements in management and therapeutic strategies, the prognosis remains poor for patients with metastatic or advanced-stage LUAD [3,4]. Research shows that changes in gene expression are associated with LUAD progression and patient prognosis [5–11], highlighting the importance of gene expression profiles in guiding the development of new therapies for LUAD. Thus, a need exists to identify new biomarkers to better understand the disease and develop effective therapies.

Research into genes related to sphingolipid metabolism holds promise for identifying new biomarkers for LUAD. The recent identification and cloning of key enzymes in this pathway have significantly advanced our understanding of sphingolipid regulation [12]. Altered enzyme expression or activity plays a crucial role in regulating cancer signaling and treatment responses [13]. Sphingolipid metabolism, through bioactive lipids like sphingosine-1-phosphate (S1P) [14], influences cancer biology by affecting the tumor microenvironment and promoting cell motility and invasion, thereby accelerating metastatic processes [15–19]. Disialoganglioside (GD3) synthase (GD3S), a key enzyme in ganglioside biosynthesis, is involved in epithelial-to-mesenchymal transition (EMT), contributing to the acquisition of more aggressive cancer cell characteristics [20]. In addition, Acid ceramidase (ASAH1) further promotes a pro-tumor sphingolipid profile by degrading ceramide into sphingosine, thereby enhancing cancer cell survival, invasiveness, and resistance to therapy [21]. Defining sphingolipid metabolism pathways and the underlying mechanisms may help identify novel biomarkers and improve LUAD therapies.

Recent studies highlight the importance of sphingolipid metabolism biomarkers, yet their role in LUAD is poorly understood. This study aimed to evaluate sphingolipid metabolism-related genes in LUAD and their impact on disease progression. The findings offer valuable insights that can inform future LUAD research and the development of targeted therapeutic strategies.

## Materials and methods

### Data acquisition and identifying differentially expressed genes

**Fig 1:**
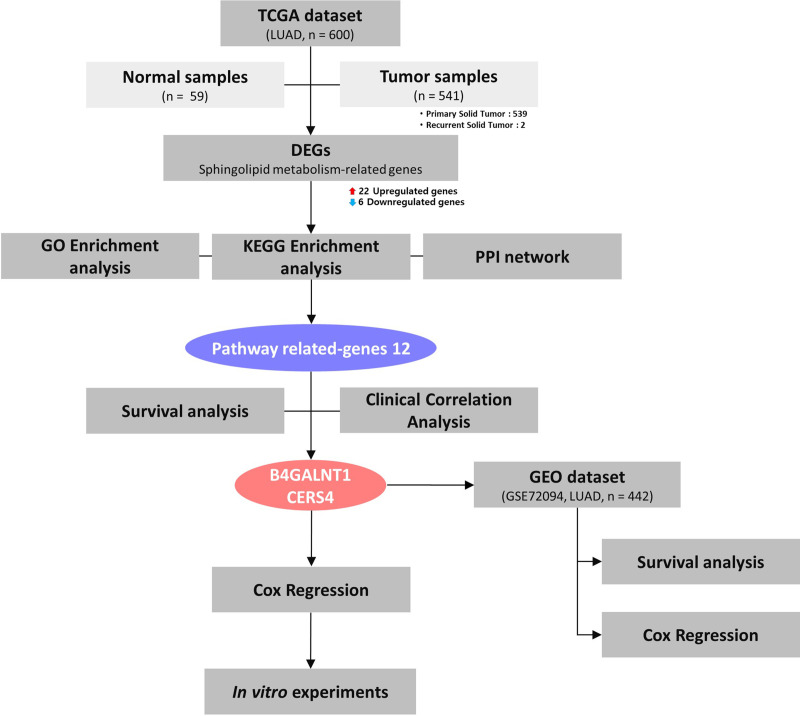
Flowchart of the analysis in the current study. LUAD gene expression data obtained from TCGA dataset were evaluated via DEG analysis to assess the differential mRNA expression of sphingolipid metabolism-related genes between NSTs and LUAD tissues. GO and KEGG enrichment analyses, as well as PPI network construction were performed to examine the biological functions correlated with the mRNAs and the interactions between the genes. Survival analysis and clinical correlation analysis were performed for pathway-related genes. The two significant genes, *B4GALNT1* and *CERS4*, were further evaluated using Cox regression analyses, and their reproducibility was validated in an independent cohort. Cell-based functional experiments were performed to assess the biological roles of *B4GALNT1* and *CERS4*. (Abbreviations: LUAD, lung adenocarcinoma; TCGA, The Cancer Genome Atlas; DEG, differential gene expression; mRNA, messenger RNA; NST, normal solid tissue; GO, Gene Ontology; KEGG, Kyoto Encyclopedia of Genes and Genomes; PPI, protein–protein interaction.).

A flowchart summarizing the study design is shown in Fig 1. The expression matrix of the LUAD dataset, downloaded from The Cancer Genome Atlas (TCGA; https://portal.gdc.cancer.gov/) using the R package TCGAbiolinks (version 2.32.0, Bioconductor) [22], comprised 541 LUAD tumor samples (primary and recurrent solid tumors) and 59 adjacent normal samples (non-tumor tissues, NSTs). Preprocessing and normalization of the expression data were performed in the R environment using Bioconductor packages (http://www.bioconductor.org/) [22]. Differentially expressed genes (DEGs) were identified using the limma package (version 3.58.1, Bioconductor) [23], with the criteria of |log2 fold change| > 1 and *P* < 0.05. The expression matrix of the GSE72094 dataset, downloaded from the Gene Expression Omnibus (GEO; https://www.ncbi.nlm.nih.gov/geo/) using the R package GEOquery (version 2.58.0, Bioconductor), comprised 442 LUAD tumor samples with corresponding clinical annotations. The normalized series matrix file provided by GEO was used, and no additional normalization was required. Expression, phenotype, and feature data were extracted from the ExpressionSet (version 2.62.0, Biobase, Bioconductor) object for downstream analyses. Clinical information for the patients is summarized in S1 and S2 Tables. This study used publicly available and fully anonymized data from TCGA. The use of public data was approved for research purposes, and informed consent was waived by the institutional review board due to the retrospective nature of the analysis. The GSE72094 dataset was also obtained from the GEO database as a publicly accessible and de-identified resource, and therefore no additional ethical approval or patient consent was required for its use.

### Functional enrichment analysis

Gene Ontology (GO) and Kyoto Encyclopedia of Genes and Genomes (KEGG) enrichment analyses were performed using the clusterProfiler R package (version 4.10.1, Bioconductor) [24] to identify genes functionally associated with biological pathways. For GO analysis, enrichment was conducted across three major domains: Biological Process (BP), Cellular Component (CC), and Molecular Function (MF). GO terms with adjusted *P*-values < 0.05 were considered significant. The top five enriched terms from each domain were visualized, and the genes associated with these representative terms were selected for further analysis. For KEGG analysis, the same gene set was used to assess pathway enrichment. Significantly enriched KEGG pathways (adjusted *P*-value < 0.05) were identified, and the corresponding genes were subjected to protein–protein interaction (PPI) network construction and survival analysis.

### PPI network analysis

To examine molecular interactions among sphingolipid metabolism-related genes, a PPI network was constructed based on data from the STRING database (https://string-db.org/) [25]. The interaction results were imported into Cytoscape software (version 3.10.3) for visualization [26]. To enhance clarity, disconnected nodes were excluded from the network. Genes forming a highly interconnected module were identified and highlighted as a putative core regulatory cluster for subsequent biological interpretation.

### Survival analysis

The prognostic significance of sphingolipid metabolism-related genes in LUAD was evaluated using Kaplan–Meier survival analysis. For each gene, patients were stratified into high-expression and low-expression groups based on optimal cutoff values, determined using the Cutoff Finder tool [27]. The cutoff values applied in the TCGA cohort were *ASAH1* = 6172, *B4GALNT1* = 135.5, *CERS6* = 1829, *CERS4* = 697.5, *SPTLC1* = 6516, *CERS5* = 1065, *GBA* = 2519, *GALC* = 3938, *CERS1* = 62.5, *SPHK1* = 2616, *UGT8* = 1128, and *SPTLC2* = 9071. In the GSE72094 validation cohort, cutoff values of 4.77 for *B4GALNT1* and 5.074 for *CERS4* were used. Survival differences between groups were assessed by the log-rank test. Kaplan–Meier survival curves were generated using the R package survminer (version 0.5.0) [28], and genes with *P*-values < 0.05 were considered statistically significant.

### Tumor stage and metastatic status analysis

The associations between sphingolipid metabolism-related gene expression and tumor stage (Stage I–IV) and metastatic status (M0 vs. M1) were assessed by integrating mRNA expression data with corresponding clinical annotations from TCGA to determine their relevance to clinical classification in LUAD. Statistical comparisons across cancer stages were performed using one-way analysis of variance (ANOVA), while statistical significance between two groups (M0 vs. M1) was assessed using Student’s t-test. Group-wise expression patterns visualized using the ggplot2 package (version 3.5.2) [29].

### Cox regression analysis

Cox proportional hazards regression analysis was performed to assess the prognostic value of *B4GALNT1* and *CERS4* expression in LUAD. Both univariate and multivariable Cox models were constructed, and the same covariates were included in each analysis. The covariates were age, gender, tumor stage (I–IV), *B4GALNT1*, *CERS4*, and mutation status of *EGFR*, *KRAS*, and *TP53*. Overall survival (OS) was defined as the time from diagnosis to death from any cause or to the last follow-up for censored cases. Hazard ratios (HRs) with 95% confidence intervals (CIs) were estimated, and variables with a two-sided *P* < 0.05 were considered statistically significant. In all analyses, stage I, female gender, and patients without *EGFR*, *KRAS*, or *TP53* mutations (wild-type) were used as the reference categories. Age was entered as a continuous variable, whereas gender, tumor stage, and mutation status were treated as categorical variables. All Cox regression analyses were performed in R using the survival package (version 3.5–8) [30].

### Cell culture

The A549 human LUAD cell line was obtained from the American Type Culture Collection (Manassas, VA, USA) and cultured in Roswell Park Memorial Institute (RPMI)-1640 medium (WELGENE, Gyeongsan, Korea) supplemented with 10% heat-inactivated fetal bovine serum (FBS; WELGENE), 2 mM L-glutamine, 100 μg/mL streptomycin, and 100 μg/mL penicillin. The cells were incubated in a humidified chamber at 37°C with 95% air and 5% CO_2_.

### Plasmid transfection and siRNA-mediated knockdown

The pcDNA3.1-CerS4-HA plasmid was kindly provided by Prof. Anthony H. Futerman (Weizmann Institute of Science, Israel). The plasmid used for overexpression experiments was constructed as previously described [31]. A549 cells were transiently transfected with 4 μg of the plasmid using Lipofectamine™ 2000 Transfection Reagent (Invitrogen, Carlsbad, CA, USA) according to the manufacturer’s instructions. For gene knockdown experiments, A549 cells were transfected with B4GALNT1-targeting siRNA duplexes (Cell Signaling Technology, Danvers, MA, USA) using Lipofectamine™ RNAiMAX (Invitrogen) following the manufacturer’s instructions. Following transfection, cells were incubated for 4–6 h, after which the medium was replaced with fresh complete medium and the cells were used for subsequent assays.

### Cell proliferation assay

Transfected A549 cells were seeded into 96-well plates (3,000 cells per well) and incubated for 24 or 48 h. To assess cell proliferation, 20 μL of XTT reagent was added to each well according to the manufacturer’s instructions (WELGENE), and the plates were incubated at 37°C for 3 h. Absorbance was measured at 450 nm using a microplate reader (Infinite F200 Pro, TECAN).

### Wound healing assay

Transfected A549 cells were cultured in RPMI-1640 medium supplemented with 10% FBS for 24 h prior to scratching. Linear wounds were generated using sterile pipette tips, and wound closure was monitored at 0, 24, and 48 h using phase-contrast microscopy. The extent of wound closure was quantified using ImageJ software.

### Western blotting analysis

Cellular lysates were obtained by resuspending 5 × 10⁵ cells in 100 μL of lysis buffer, containing 0.1 mM sodium orthovanadate, 100 μM phenylmethylsulfonyl fluoride, 25 mM 3-(N-morpholino) propanesulfonic acid, 0.1% Triton X-100, 15 mM MgCl₂, 137 mM NaCl, 15 mM ethylene glycol tetraacetic acid, and 20 μM leupeptin, with the pH adjusted to 7.2. Cells were lysed via sonication and incubated at 4°C for 30 min. After centrifuging at 10,000 × g and 4°C for 15 min, the supernatant fractions were collected. Proteins were separated using sodium dodecyl sulfate–polyacrylamide gel electrophoresis (SDS–PAGE) to analyze approximately 30 μg of protein. Proteins were electrotransferred to Immobilon-P membranes (Millipore, Billerica, MA, USA) and incubated with a blocking solution containing 0.05% Tween® 20 and 5% nonfat dry milk in Tris-buffered saline (TBS) for 3 h. After three washes with TBS containing Tween® 20, the membranes were exposed to primary antibodies overnight. The following primary antibodies were used: anti‑β‑actin (A5441, 1:10,000), anti‑HA (H6908, 1:1,000), anti‑CerS4 (SAB4301210, 1:1,000), and anti‑B4GALNT1 (SAB1410478, 1:700) from Sigma‑Aldrich (St. Louis, MO, USA); anti‑vimentin (5741, 1:700) and anti‑Snail (3879, 1:700) from Cell Signaling Technology (Danvers, MA, USA); and anti‑E‑cadherin (sc‑71008, 1:700) and anti‑N‑cadherin (sc‑59987, 1:700) from Santa Cruz Biotechnology (Dallas, TX, USA). An ECL western blotting kit (Millipore) was used to detect specific proteins according to the manufacturer’s instructions. Signal intensity was analyzed using the Chemi Image documentation system (Fusion Rs7; VILBER LOUTMAT, Collégien, France).

### Statistical analysis

All statistical analyses were performed using R software (version 4.3.3, R Foundation for Statistical Computing, Vienna, Austria). Statistical significance was set at *P*-value < 0.05. Continuous variables that were normally distributed were analyzed using Student’s *t*-*t*est. One-way ANOVA was applied to compare continuous variables across more than two groups, and pairwise comparisons were performed using unadjusted Student’s *t*-*t*ests. No post-hoc multiple comparison correction was applied.

## Results

### Differentially expressed sphingolipid metabolism-related genes in LUAD

**Fig 2:**
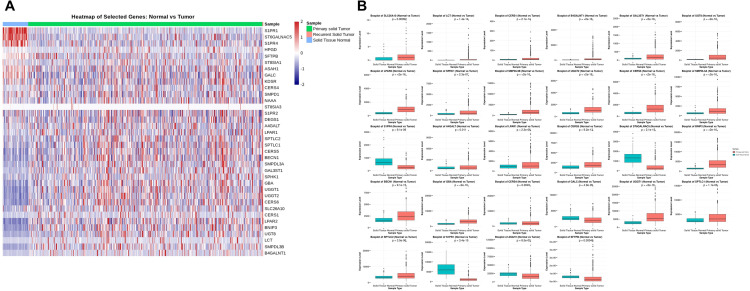
Differentially expressed sphingolipid metabolism-related genes. **(A)** Heatmap of significantly differentially expressed sphingolipid metabolism-related genes based on mRNA expression levels between LUAD tissues and NSTs. In the matrix format, rows correspond to individual sphingolipid metabolism-related genes; columns represent distinct tissue samples. Each cell in the matrix displays the relative mRNA expression of a specific gene in a particular tissue. Red and blue indicate higher and lower expression levels, respectively. Samples are organized from NSTs to LUAD tissues based on the standardized expression levels of each gene. **(B)** Relative mRNA expression changes of various sphingolipid metabolism-related genes; statistical significance set at *P* < 0.05 (NSTs vs. LUAD tissues). (Abbreviations: LUAD, lung adenocarcinoma; NST, normal solid tissue; mRNA, messenger RNA.).

The expression levels of mRNA related to sphingolipid metabolism were analyzed and compared between NSTs, and LUAD tissues using data from TCGA LUAD dataset (Fig 2A). Out of the 36 genes related to sphingolipid metabolism, 22 were significantly upregulated, and 6 genes were significantly downregulated in LUAD tissues compared to NSTs (Fig 2B). Meanwhile, the expression of eight genes did not differ significantly between the two groups (S1 Fig).

### Functional enrichment and hub gene network analysis of sphingolipid pathway regulators

**Fig 3:**
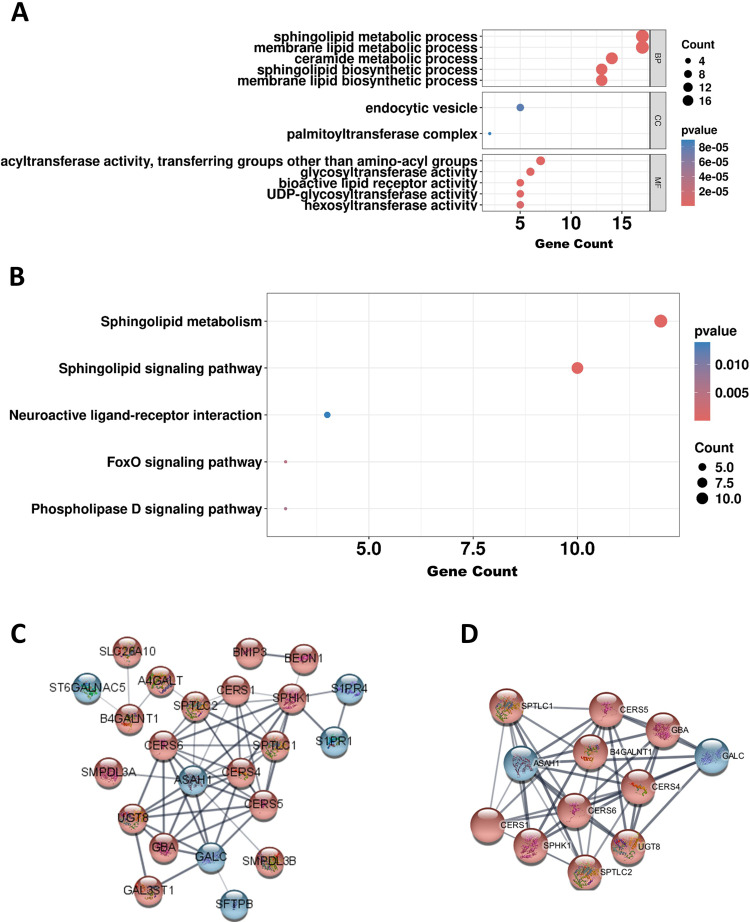
Functional enrichment analyses and PPI network construction. **(A)** GO enrichment analysis of biological process, cellular component, and molecular function terms. The top five significantly enriched terms from each domain are visualized. **(B)** Top five significantly enriched KEGG pathways based on enrichment analysis. **(C)** PPI network constructed with genes involved in the sphingolipid metabolism pathway identified in the KEGG analysis. **(D)** From the PPI network, genes with high interconnectivity were identified as a core module. Nodes represent genes, and edges indicate known or predicted interactions. (Abbreviations: GO, Gene Ontology; KEGG, Kyoto Encyclopedia of Genes and Genomes; PPI, protein–protein interaction.).

GO analysis revealed significant enrichment in BPs such as sphingolipid metabolism, membrane lipid metabolism, and ceramide metabolic process. Enriched CC terms included endocytic vesicle and palmitoyltransferase complex, while MF terms were associated with acyltransferase activity, transferring non-amino-acyl groups, glycosyltransferase activity, and bioactive lipid receptor activity (Fig 3A; S3 Table). KEGG pathway analysis revealed significant enrichment of sphingolipid metabolism and sphingolipid signaling pathways, suggesting their potential involvement in LUAD pathogenesis (Fig 3B; S4 Table).

To further explore the interactions among genes related to sphingolipid metabolism, a PPI network was constructed using the STRING database. Based on 23 genes successfully mapped to KEGG pathways from the 28sphingolipid metabolism-related DEGs (22 upregulated and 6 downregulated genes), the network revealed myriad interactions, suggesting that these genes may function cooperatively within shared biological pathways or regulatory modules (Fig 3C). Furthermore, 12 genes formed a highly interconnected core within the network (Fig 3D), indicating that they may serve as central components in sphingolipid-related molecular mechanisms.

### Prognostic biomarkers among core sphingolipid metabolism-related genes

**Fig 4:**
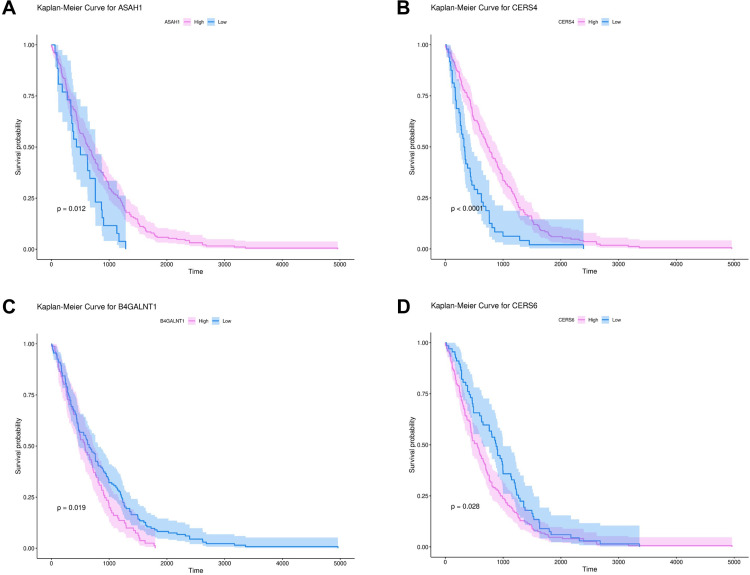
Survival analysis of key sphingolipid metabolism-related genes. **(A–D)** Kaplan–Meier survival curves for *ASAH1*, *CERS4*, *B4GALNT1*, and *CERS6*, created using a non-parametric log-rank test. For each gene, patients are stratified into high-expression and low-expression groups based on the optimal cutoff value. The x-axis represents survival time (days); the y-axis indicates the survival probability. Shaded areas represent 95% confidence intervals for each group. (Abbreviations: *ASAH1*, N-acylsphingosine amidohydrolase 1; *CERS4*, ceramide synthase 4; *B4GALNT1*, beta-1,4-N-acetyl-galactosaminyltransferase 1; *CERS6*, ceramide synthase 6.).

The prognostic significance of 12 core genes was predicted to play central roles in the sphingolipid metabolism network. Patients with LUAD were assigned to the high-expression and low-expression groups for each of the 12 core sphingolipid metabolism genes and Kaplan–Meier survival analyses was performed with log-rank testing. Among the 12 genes, overall survival was significantly correlated with *ASAH1*, *CERS4*, *B4GALNT1*, and *CERS6* expression (Fig 4). Downregulation of *ASAH1* and *CERS4* (Figs 4A and 4B) and upregulation of *B4GALNT1* and *CERS6* significantly correlated with poor overall survival (Figs 4C and 4D). No significant correlations were observed for the remaining eight genes with overall survival (S2 Fig). These findings thus refined the study focus to *ASAH1*, *CERS4*, *B4GALNT1* and *CERS6* as the most prognostically relevant sphingolipid metabolism-related genes in LUAD.

### Cancer stage and metastasis-associated sphingolipid biomarkers in LUAD

**Fig 5:**
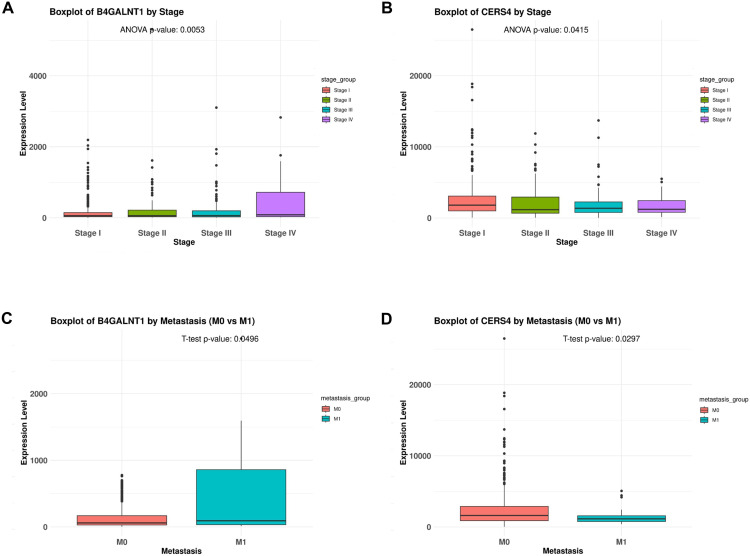
Expression of *B4GALNT1* and *CERS4* based on tumor stage and metastatic status. **(A, B)** mRNA expression levels of *B4GALNT1* and *CERS4* across different tumor stages (Stage I, II, III, and IV) in LUAD. **(C, D)** mRNA expression levels of *B4GALNT1* and *CERS4* according to metastatic status (M0 vs. M1). Statistical significance assessed using one-way ANOVA for stage comparison and Student’s *t*-test for metastasis comparison. (Abbreviations: LUAD, lung adenocarcinoma; mRNA, messenger RNA; ANOVA, analysis of variance; *B4GALNT1*, beta-1,4-N-acetyl-galactosaminyltransferase 1; *CERS4*, ceramide synthase 4.).

The mRNA expression levels of four key genes—*ASAH1*, *B4GALNT1*, *CERS6*, and *CERS4*—were assessed across tumor stages (I–IV) and metastatic statuses (M0 vs. M1; M0, no distant metastasis; M1, distant metastasis). Significant changes were observed in *B4GALNT1* and *CERS4* expression based on cancer stage and metastatic status (Fig 5). Compared with early stages, *B4GALNT1* was upregulated and *CERS4* was downregulated in advanced stages (stage IV) (Fig 5A: *P* = 0.0053; Fig 5B: *P* = 0.0415). Regarding metastatic status, *B4GALNT1* was upregulated (Fig 5C: *P* = 0.0496) and *CERS4* was downregulated in M1 compared with M0 (Fig 5D: *P* = 0.0297). No significant correlations were observed for *ASAH1* and *CERS6* (S3 Fig).

### Prognostic significance of *B4GALNT1* and *CERS4* expression in LUAD

**Fig 6:**
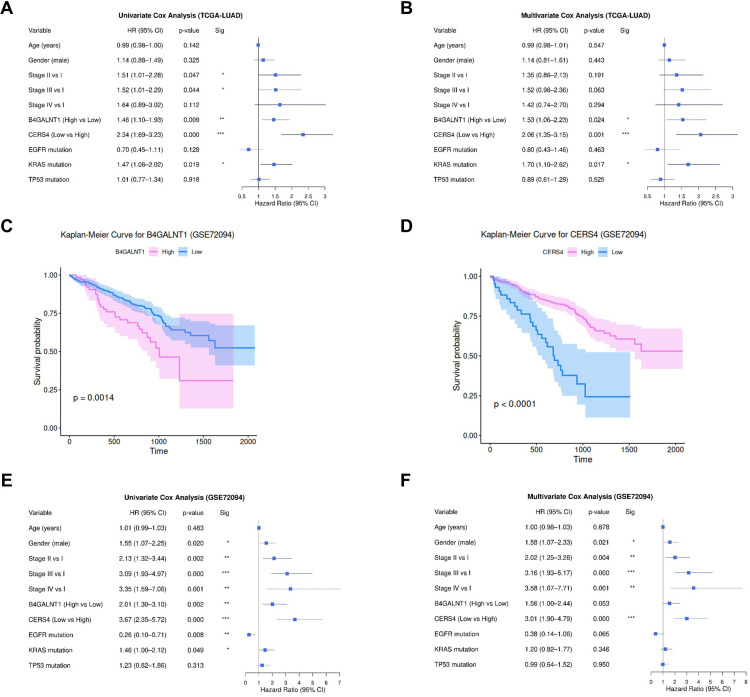
Prognostic significance of *B4GALNT1* and *CERS4* expression in LUAD. **(A, B)** Cox regression analyses of *B4GALNT1* and *CERS4* expression in the TCGA-LUAD dataset. **(C, D)** Kaplan–Meier survival analyses of *B4GALNT1* and *CERS4* expression in the GSE72094 dataset. **(E, F)** Cox regression analyses of *B4GALNT1* and *CERS4* expression in the GSE72094 dataset. (Abbreviations: LUAD, lung adenocarcinoma; HR, hazard ratio; CI, confidence interval; B4GALNT1, beta-1,4-N-acetyl-galactosaminyltransferase 1; CERS4, ceramide synthase 4.).

Cox regression analysis was performed to evaluate the prognostic value of *B4GALNT1* and *CERS4* expression (Figs 6A and 6B). Patients with high *B4GALNT1* expression showed significantly poor survival compared with those with low expression (HR = 1.46, 95% CI = 1.10–1.93, *P* = 0.009). In contrast, patients with low *CERS4* expression exhibited significantly poor overall survival compared with those with high expression (HR = 2.34, 95% CI = 1.69–3.23, *P* < 0.0001). In the multivariable analysis adjusted for age, gender, tumor stage, and mutation status of *EGFR*, *KRAS*, and *TP53*, high *B4GALNT1* expression remained significantly correlated with poor overall survival (HR = 1.53, 95% CI = 1.06–2.23, *P* = 0.024), and low *CERS4* expression remained significantly associated with poor overall survival (HR = 2.06, 95% CI = 1.35–3.15, *P* = 0.001).

The prognostic significance of *B4GALNT1* and *CERS4* was evaluated in the validation dataset GSE72094. Kaplan–Meier survival analyses showed that high *B4GALNT1* expression was significantly associated with poor overall survival (Fig 6C: *P* = 0.0014), whereas low *CERS4* expression was significantly associated with poor overall survival (Fig 6D: *P* < 0.0001,). Cox regression analysis was performed in the GSE72094 dataset to evaluate the prognostic value of *B4GALNT1* and *CERS4* expression. Patients with high *B4GALNT1* expression showed significantly poor survival compared with those with low expression (HR = 2.01, 95% CI = 1.30–3.10, *P* = 0.002). In contrast, patients with low *CERS4* expression exhibited significantly poor overall survival compared with those with high expression (HR = 3.67, 95% CI = 2.35–5.72, *P* < 0.0001). In the multivariable analysis adjusted for age, gender, tumor stage, and mutation status of *EGFR*, *KRAS*, and *TP53*, high *B4GALNT1* expression showed a borderline correlation with poor overall survival (HR = 1.56, 95% CI = 1.00–2.44, *P* = 0.053), whereas low *CERS4* expression remained significantly associated with poor overall survival (HR = 3.01, 95% CI = 1.90–4.79, *P* < 0.0001).

### *In vitro* functional validation of B4GALNT1 and CERS4 as key regulators in A549 cells

**Fig 7:**
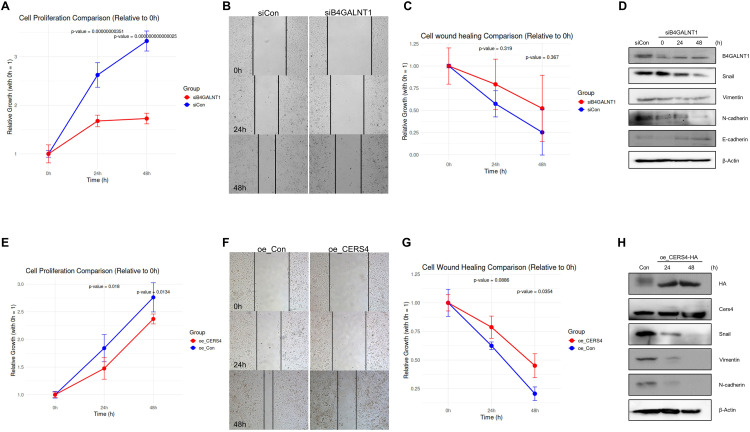
Effect of *B4GALNT1* knockdown and *CERS4* overexpression on A549 cell aggressiveness. **(A)** Relative growth at 24 h and 48 h compared to 0 h in siCon and siB4GALNT1 groups. **(B)** Representative bright-field images of the wound area 0, 24, and 48 h after scratch in siCon and siB4GALNT1 groups. **(C)** Relative wound closure at 24 h and 48 h compared to 0 h in siCon and siB4GALNT1 groups. **(D)** Western blot analysis of EMT markers (Snail, Vimentin, N-cadherin, E-cadherin) and B4GALNT1 at 0, 24, and 48 h after siB4GALNT1 treatment; β-actin is the loading control. **(E)** Relative cell proliferation at 24 h and 48 h compared to 0 h in oe_Con and oe_CERS4 groups. **(F)** Representative bright-field images of the wound area at 0, 24, and 48 h after scratch in oe_Con and oe_CERS4 groups. **(G)** Relative wound closure at 24 h and 48 h compared to 0 h in oe_Con and oe_CERS4 groups. **(H)** Western blot analysis of EMT markers (Snail, Vimentin, N-cadherin) and CERS4 at 0, 24, and 48 h after oe_CERS4-HA transfection; HA and β-Actin were also detected. (Abbreviations: EMT, epithelial–mesenchymal transition; siCon, small interfering RNA control; siB4GALNT1, siRNA targeting *B4GALNT1*; oe_Con, overexpression control; oe_*CERS4*, overexpression of *CERS4*; HA, hemagglutinin tag; *B4GALNT1*, beta-1,4-N-acetyl-galactosaminyltransferase 1; *CERS4*, ceramide synthase 4.).

Based on their significant differential expression across tumor stages and metastatic statuses in LUAD, *CERS4* and *B4GALNT1* were selected for subsequent *in vitro* functional analyses in the A549 cell line. The impact of *B4GALNT1* knockdown and *CERS4* overexpression was assessed on cell proliferation, migration, and EMT. Silencing *B4GALNT1* in A549 cells by siRNA significantly reduced cell viability at 24 h and 48 h post-transfection compared with the control (Fig 7A). In wound-healing assays, siB4GALNT1 cells exhibited a trend toward delayed wound closure at 24 h and 48 h (Figs 7B and 7C). Western blot analysis confirmed reduced B4GALNT1 protein levels and modulated expression of EMT markers: E-cadherin was upregulated at 24 h and 48 h, while vimentin, Snail, and N-cadherin levels were progressively decreased relative to control cells (Fig 7D), indicating inhibition of the EMT pathway.

Furthermore, overexpression of *CERS4* (HA-tagged) in A549 cells led to significant decreases in viability at 24 h and 48 h compared with control cells (Fig 7E). In wound-healing assays, oe_CERS4 cells exhibited a significant delay in wound closure at 24 h and 48 h (Figs 7F and 7G), demonstrating impaired migratory behavior. Western blotting verified increased CERS4 protein expression and markedly reduced Snail, vimentin, and N-cadherin levels over time in oe_CERS4 cells versus control. These results further supported suppression of the EMT process upon CERS4 gain of function (Fig 7H).

## Discussion

Sphingolipid metabolism has gained increasing attention as a critical area of cancer research, particularly for its therapeutic implications [32]. Accordingly, sphingolipid-based strategies are emerging as promising approaches for chemotherapy and immunotherapy. Alterations in sphingolipid metabolism genes, such as *SPHK1*, are significantly associated with patient prognosis, supporting their potential as therapeutic targets [33–52]. This study elucidates how changes in genes related to sphingolipid metabolism correlate with overall transcriptomic changes in cancer progression and prognosis.

Genes related to the sphingolipid metabolic pathway were identified and evaluated as potential candidate biomarkers. Two genes, namely *B4GALNT1* and *CERS4*, were identified as key regulators of LUAD progression and patient prognosis. Both genes demonstrated significant differential expression based on tumor stage and metastatic status and correlated with overall survival in LUAD. *B4GALNT1*, which encodes the ganglioside GM2/GD2 synthase, was markedly upregulated while *CERS4—*a ceramide synthase—was downregulated in advanced-stage tumors and in cases with distant metastasis. Correspondingly, Kaplan–Meier survival analyses revealed that high *B4GALNT1* expression and low *CERS4* expression correlated with poorer overall survival. These correlations were further supported by Cox proportional hazards regression analyses in the TCGA-LUAD cohort, where high *B4GALNT1* expression and low *CERS4* expression remained significantly correlated with poor overall survival after adjustment for age, gender, tumor stage, and major driver mutations (*EGFR*, *KRAS*, *TP53*). Importantly, consistent prognostic patterns were reproduced in the independent GSE72094 validation dataset, in which high *B4GALNT1* expression and low *CERS4* expression consistently stratified patient survival. The reproducibility of these findings across two large cohorts indicates that the prognostic impact of *B4GALNT1* and *CERS4* is unlikely to be due to confounding by stage or mutation status, but rather reflects their intrinsic contribution to LUAD progression. Taken together, the results of the current study suggest that *B4GALNT1* functions as a pro-tumorigenic factor in LUAD, whereas *CERS4* may act as a tumor-suppressive factor, both serving as valuable prognostic biomarkers for disease outcome.

Our data align with and extend prior studies implicating sphingolipid-related enzymes in cancer progression and therapy response. Notably, *B4GALNT1* promotes LUAD invasion and metastasis by activating the JNK/c-Jun/Slug signaling pathway. In clinical LUAD samples, *B4GALNT1* expression correlates with advanced tumor stage, lymph node metastasis, and decreased patient survival [53], agreeing with our observation that *B4GALNT1* is elevated in stage IV and metastatic LUAD and predicts poor prognosis. Similarly, we previously reported that high *B4GALNT1* levels correlate with adverse patient outcomes in hepatocellular carcinoma [54], suggesting that the oncogenic role of *B4GALNT1* may extend to multiple cancer types. Mechanistically, *B4GALNT1* overexpression drives the synthesis of complex gangliosides on the cell surface, which can modulate signaling pathways that favor cancer cell survival, motility, and EMT [53]. Our functional experiments further demonstrated that silencing *B4GALNT1* in LUAD cells significantly suppressed proliferation and migration, and partially reversed EMT, as evidenced by increased E-cadherin expression and decreased expression of mesenchymal markers (vimentin, Snail, and N-cadherin). These findings reinforce the notion that *B4GALNT1* actively sustains an aggressive phenotype in LUAD cells, likely by sustaining pro-metastatic signaling networks.

In contrast, CERS4 may act as a tumor suppressor in LUAD. We observed higher *CERS4* expression in early-stage LUAD tissues than in advanced tumors, and lower *CERS4* levels were linked to significantly poorer survival. This is consistent with previous reports suggesting that *CERS4* expression is associated with tumor-suppressive effects or favorable prognosis in certain cancers. For instance, upregulated *CERS4* in non-small cell lung carcinoma has been linked to improved patient prognosis, while loss of *CERS4* expression in colorectal cancer is associated with worse survival outcomes [55,56]. In our transcriptomic analysis, low *CERS4* correlated with more advanced disease, indicating that *CERS4* deficiency might facilitate tumor progression. Functionally, *CERS4* encodes a ceramide synthase responsible for generating long-chain ceramide bioactive sphingolipids, which promote apoptotic and anti-proliferative signaling [57]. Supporting this, experimental overexpression of *CERS4* in LUAD cells significantly reduced their viability and migratory ability, concomitant with a sharp decrease in EMT marker expression (Snail, vimentin, N-cadherin). These results align with observations in other cancer models. Notably, Hartmann et al. reported that enforced *CERS4* expression in breast and colon cancer cells leads to the accumulation of long-chain ceramides and subsequent growth arrest [58]. Genetic loss of *CERS4 in vivo* enhanced metastatic spread in a mammary tumor model, where *CERS4* knockout mice exhibited a significantly higher incidence of lung metastases than wild-type, despite similar primary tumor growth. Intriguingly, *CERS4* loss in this mouse model was also linked to a more immunosuppressive tumor microenvironment, including increased PD-L1 signaling and regulatory T-cell infiltration [59]. Such findings underscore the multifaceted role of *CERS4* in restraining tumor progression and maintaining immune surveillance. Indeed, a recent study in patients with non-small cell lung carcinoma found that high *CERS4* expression correlates with improved responses to anti–PD-1 immunotherapy, presumably by modulating the balance of exhausted T cells in the tumor microenvironment [56]. Together with our results, these data highlight *CERS4* as a clinically significant enzyme whose downregulation in LUAD may promote intrinsic tumor growth and dampen anti-tumor immune responses.

Collectively, our study suggests that *B4GALNT1* and *CERS4* may serve as prognostic biomarkers and potential therapeutic targets in LUAD. *B4GALNT1* appears to promote malignant progression by enhancing cell proliferation, migration, and EMT, which aligns with its involvement in pro-tumorigenic ganglioside synthesis and the activation of EMT-related transcription factors [53]. Thus, *B4GALNT1* upregulation could serve as a warning indicator of aggressive, metastatic disease, as evidenced by its strong correlation with advanced stage, distant metastasis, and poor patient survival in the LUAD cohort. Targeting *B4GALNT1* or its downstream gangliosides may represent a potential strategy to impede LUAD progression. In contrast, *CERS4* generates ceramide species that tend to inhibit cell growth and spread, and its loss removes an important brake on tumor progression [58]. This is analogous to the “sphingolipid rheostat” model, in which ceramide accumulation induces antiproliferative and apoptotic pathways [60]. Meanwhile, *CERS4* downregulation in tumors might flag a loss of ceramide-mediated growth control and immune engagement. Recent preclinical evidence suggests that restoring ceramide synthesis or replicating its effects through ceramide analogs or modulators of sphingolipid metabolism may inhibit tumor growth and enhance immunotherapy responses [59]. However, the functional role of *CERS4* may vary depending on tumor type and cellular context, highlighting the need to further investigate the mechanistic basis of *CERS4*-mediated signaling in LUAD and other malignancies. These contrasting roles of *B4GALNT1* and *CERS4* provide important insights into the transcriptomic and phenotypic alterations observed during LUAD progression in this study.

Finally, we acknowledge certain limitations in this study. The transcriptomic data used in this study may not fully capture the global heterogeneity of LUAD patients, potentially limiting the generalizability of our findings. Additionally, our functional assays were conducted in a single cell line; hence, validation in *in vivo* systems is important. Despite these limitations, our integrated analysis strongly supports *B4GALNT1* and *CERS4* as meaningful biomarkers with regulatory significance in LUAD. Their opposing expression and functions may drive LUAD aggressiveness and worsen patient outcomes. Recognizing the prognostic value of these genes and elucidating their molecular interplay may inform future strategies for risk stratification and developing targeted therapies in LUAD.

## Conclusions

This study demonstrates that *B4GALNT1* and *CERS4* may contribute to LUAD malignancy through distinct functional mechanisms. Their expression levels significantly correlate with tumor stage, metastasis, and patient survival. Notably, to our knowledge, this is the first study to experimentally evaluate the role of *CERS4* in LUAD, providing a foundation for understanding its clinical relevance. Cumulatively, these findings underscore the potential of *B4GALNT1* and *CERS4* as biomarkers and therapeutic targets in LUAD, while highlighting the need for further investigation into their underlying molecular mechanisms.

## Supporting information

S1 Fig Analysis of sphingolipid metabolism-related genes with no significant expression differences. The graph shows the relative mRNA expression levels of sphingolipid metabolism-related genes, with eight genes showing no significant differences (*P* ≥ 0.05) when comparing LUAD tissues to NSTs.
(JPEG)

S2 Fig Survival analysis of eight genes with no significant correlation to overall survival. No significant correlations with overall survival were observed for the remaining eight sphingolipid metabolism-related genes, including *SPTLC1*, *CERS5*, *GBA*, *GALC*, *CERS1*, *SPHK1*, *UGT8*, and *SPTLC2*.
(JPEG)

S3 Fig Expression analysis of *ASAH1* and *CERS6* across cancer stages and metastatic statuses. Box plots showing the mRNA expression levels of *ASAH1* and *CERS6* across different cancer stages (Stage I, II, III, and IV) and metastatic status (M0 vs. M1) in LUAD.
(JPEG)

S4 Fig Uncropped western blot membrane image related to the original manuscript. Related to Fig 6.
(PDF)

S1 Table Clinical information of LUAD patients from TCGA dataset. (DOCX)

S2 Table Clinical information of LUAD patients from GEO dataset. (DOCX)

S3 Table GO biological process enrichment analysis results. (XLSX)

S4 Table KEGG pathway enrichment analysis results. (XLSX)

## References

[1] Thandra KC, Barsouk A, Saginala K, Aluru JS, Barsouk A. Epidemiology of lung cancer. Contemp Oncol (Pozn). 2021;25(1):45–52. doi: 10.5114/wo.2021.103829 33911981 PMC8063897

[2] Herbst RS, Morgensztern D, Boshoff C. The biology and management of non-small cell lung cancer. Nature. 2018;553(7689):446–54. doi: 10.1038/nature25183 29364287

[3] Kuhn E, Morbini P, Cancellieri A, Damiani S, Cavazza A, Comin CE. Adenocarcinoma classification: patterns and prognosis. Pathologica. 2018;110(1):5–11. 30259909

[4] Li Y, Yan B, He S. Advances and challenges in the treatment of lung cancer. Biomed Pharmacother. 2023;169:115891. doi: 10.1016/j.biopha.2023.115891 37979378

[5] Zhao M, Li M, Chen Z, Bian Y, Zheng Y, Hu Z, et al. Identification of immune-related gene signature predicting survival in the tumor microenvironment of lung adenocarcinoma. Immunogenetics. 2020;72(9–10):455–65. doi: 10.1007/s00251-020-01189-z 33188484

[6] Li N, Wang J, Zhan X. Identification of immune-related gene signatures in lung adenocarcinoma and lung squamous cell carcinoma. Front Immunol. 2021;12:752643. doi: 10.3389/fimmu.2021.752643 34887858 PMC8649721

[7] Zhao R, Ding D, Ding Y, Han R, Wang X, Zhu C. Predicting differences in treatment response and survival time of lung adenocarcinoma patients based on a prognostic risk model of glycolysis-related genes. Front Genet. 2022;13:828543. doi: 10.3389/fgene.2022.828543 35692818 PMC9174756

[8] Lin YY, Wang YC, Yeh DW, Hung CY, Yeh YC, Ho HL, et al. Gene expression profile in primary tumor is associated with brain-tropism of metastasis from lung adenocarcinoma. Int J Mol Sci. 2021;22(24). 10.3390/ijms222413374 PMC8703941 34948172

[9] Koh YW, Han J-H, Haam S, Lee HW. An immune-related gene expression signature predicts brain metastasis in lung adenocarcinoma patients after surgery: gene expression profile and immunohistochemical analyses. Transl Lung Cancer Res. 2021;10(2):802–14. doi: 10.21037/tlcr-20-1056 33718023 PMC7947384

[10] Hwang SH, Kim J. Comprehensive analysis of chromobox 1 expression, DNA methylation and non-coding RNA interactions in lung adenocarcinoma. 2025.

[11] Gwon E-J, Bae A-N, Park J-H, Lee J-H. EXO1 is a potential prognostic biomarker in lung cancer. anatomy & biological anthropology. 2025;38(2):123–8.

[12] Ogretmen B. Sphingolipid metabolism in cancer signalling and therapy. Nat Rev Cancer. 2018;18(1):33–50. doi: 10.1038/nrc.2017.96 29147025 PMC5818153

[13] Ogretmen B, Hannun YA. Biologically active sphingolipids in cancer pathogenesis and treatment. Nat Rev Cancer. 2004;4(8):604–16. doi: 10.1038/nrc1411 15286740

[14] Wigger D, Gulbins E, Kleuser B, Schumacher F. Monitoring the sphingolipid de novo synthesis by stable-isotope labeling and liquid chromatography-mass spectrometry. Front Cell Dev Biol. 2019;7:210. doi: 10.3389/fcell.2019.00210 31632963 PMC6779703

[15] Zeng S, Liang Y, Hu H, Wang F, Liang L. Endothelial cell-derived S1P promotes migration and stemness by binding with GPR63 in colorectal cancer. Pathol Res Pract. 2022;240:154197. doi: 10.1016/j.prp.2022.154197 36371997

[16] Liang J, Nagahashi M, Kim EY, Harikumar KB, Yamada A, Huang W-C, et al. Sphingosine-1-phosphate links persistent STAT3 activation, chronic intestinal inflammation, and development of colitis-associated cancer. Cancer Cell. 2013;23(1):107–20. doi: 10.1016/j.ccr.2012.11.013 23273921 PMC3578577

[17] Zhao S, Li J. Sphingosine-1-phosphate induces the migration of thyroid follicular carcinoma cells through the microRNA-17/PTK6/ERK1/2 pathway. PLoS One. 2015;10(3):e0119148. doi: 10.1371/journal.pone.0119148 25748447 PMC4351951

[18] Zheng X, Li W, Ren L, Liu J, Pang X, Chen X, et al. The sphingosine kinase-1/sphingosine-1-phosphate axis in cancer: Potential target for anticancer therapy. Pharmacol Ther. 2019;195:85–99. doi: 10.1016/j.pharmthera.2018.10.011 30347210

[19] Spiegel S, Milstien S. The outs and the ins of sphingosine-1-phosphate in immunity. Nat Rev Immunol. 2011;11(6):403–15. doi: 10.1038/nri2974 21546914 PMC3368251

[20] Levade T, Andrieu-Abadie N, Micheau O, Legembre P, Ségui B. Sphingolipids modulate the epithelial-mesenchymal transition in cancer. Cell Death Discov. 2015;1:15001. doi: 10.1038/cddiscovery.2015.1 27551437 PMC4979435

[21] Reddi KK, Chava S, Chabattula SC, Edwards YJK, Singh K, Gupta R. ASAH1 facilitates TNBC by DUSP5 suppression-driven activation of MAP kinase pathway and represents a therapeutic vulnerability. Cell Death Dis. 2024;15(6):452. doi: 10.1038/s41419-024-06831-2 38926346 PMC11208621

[22] Gentleman RC, Carey VJ, Bates DM, Bolstad B, Dettling M, Dudoit S, et al. Bioconductor: open software development for computational biology and bioinformatics. Genome Biol. 2004;5(10):R80. doi: 10.1186/gb-2004-5-10-r80 15461798 PMC545600

[23] Ritchie ME, Phipson B, Wu D, Hu Y, Law CW, Shi W, et al. limma powers differential expression analyses for RNA-sequencing and microarray studies. Nucleic Acids Res. 2015;43(7):e47. doi: 10.1093/nar/gkv007 25605792 PMC4402510

[24] Yu G, Wang L-G, Han Y, He Q-Y. clusterProfiler: an R package for comparing biological themes among gene clusters. OMICS. 2012;16(5):284–7. doi: 10.1089/omi.2011.0118 22455463 PMC3339379

[25] Szklarczyk D, Kirsch R, Koutrouli M, Nastou K, Mehryary F, Hachilif R, et al. The STRING database in 2023: protein-protein association networks and functional enrichment analyses for any sequenced genome of interest. Nucleic Acids Res. 2023;51(D1):D638–46. doi: 10.1093/nar/gkac1000 36370105 PMC9825434

[26] Bader GD, Hogue CWV. An automated method for finding molecular complexes in large protein interaction networks. BMC Bioinformatics. 2003;4:2. doi: 10.1186/1471-2105-4-2 12525261 PMC149346

[27] Budczies J, Klauschen F, Sinn BV, Győrffy B, Schmitt WD, Darb-Esfahani S, et al. Cutoff Finder: a comprehensive and straightforward Web application enabling rapid biomarker cutoff optimization. PLoS One. 2012;7(12):e51862. doi: 10.1371/journal.pone.0051862 23251644 PMC3522617

[28] Kassambara AKM, Biecek P. Survminer: Drawing survival curves using ‘ggplot2’. R package; 2025.

[29] H W. ggplot2: Elegant Graphics for Data Analysis. New York: Springer-Verlag; 2016.

[30] Ttltaec C. Survival: Survival Analysis. CRAN; 2024.

[31] Kim S-J, Seo I, Kim MH, Park J-W, Kim S, Park W-J. Ceramide synthase 4 overexpression exerts oncogenic properties in breast cancer. Lipids Health Dis. 2023;22(1):183. doi: 10.1186/s12944-023-01930-z 37885013 PMC10605224

[32] Rufail ML, Bassi R, Giussani P. Sphingosine-1-phosphate metabolic pathway in cancer: implications for therapeutic targets. Int J Mol Sci. 2025;26(3). 10.3390/ijms26031056 PMC11817292 39940821

[33] Kim H-S, Yoon G, Ryu J-Y, Cho Y-J, Choi J-J, Lee Y-Y, et al. Sphingosine kinase 1 is a reliable prognostic factor and a novel therapeutic target for uterine cervical cancer. Oncotarget. 2015;6(29):26746–56. doi: 10.18632/oncotarget.4818 26311741 PMC4694949

[34] Li W, Yu CP, Xia J t, Zhang L, Weng GX, Zheng H q. Sphingosine kinase 1 is associated with gastric cancer progression and poor survival of patients. Clinical Cancer Research. 2009;15(4):1393–9. 19228740 10.1158/1078-0432.CCR-08-1158

[35] Gachechiladze M, Tichý T, Kolek V, Grygárková I, Klein J, Mgebrishvili G, et al. Sphingosine kinase-1 predicts overall survival outcomes in non-small cell lung cancer patients treated with carboplatin and navelbine. Oncol Lett. 2019;18(2):1259–66. doi: 10.3892/ol.2019.10447 31423186 PMC6607215

[36] Yin S, Miao Z, Tan Y, Wang P, Xu X, Zhang C, et al. SPHK1-induced autophagy in peritoneal mesothelial cell enhances gastric cancer peritoneal dissemination. Cancer Med. 2019;8(4):1731–43. doi: 10.1002/cam4.2041 30791228 PMC6488120

[37] Liu SQ, Xu CY, Wu WH, Fu ZH, He SW, Qin MB, et al. Sphingosine kinase 1 promotes the metastasis of colorectal cancer by inducing the epithelial‑mesenchymal transition mediated by the FAK/AKT/MMPs axis. Int J Oncol. 2019;54(1):41–52. 30365116 10.3892/ijo.2018.4607 PMC6254930

[38] Su YJ, Zhang JX, Li SM, Tan XH, Huang JA. Relationship of vasculogenic mimicry, SphK1 expression, and Cx43 expression to metastasis and prognosis in colorectal cancer. International Journal of Clinical and Experimental Pathology. 2018;11(11):5290. 31949609 PMC6963043

[39] Xu Y, Dong B, Wang J, Zhang J, Xue W, Huang Y. Sphingosine kinase 1 overexpression contributes to sunitinib resistance in clear cell renal cell carcinoma. Oncoimmunology. 2018;7(12):e1502130. doi: 10.1080/2162402X.2018.1502130 30524898 PMC6279321

[40] Cai H, Xie X, Ji L, Ruan X, Zheng Z. Sphingosine kinase 1: A novel independent prognosis biomarker in hepatocellular carcinoma. Oncol Lett. 2017;13(4):2316–22. doi: 10.3892/ol.2017.5732 28454397 PMC5403457

[41] Zhu Y-J, You H, Tan J-X, Li F, Qiu Z, Li H-Z, et al. Overexpression of sphingosine kinase 1 is predictive of poor prognosis in human breast cancer. Oncol Lett. 2017;14(1):63–72. doi: 10.3892/ol.2017.6134 28693136 PMC5494825

[42] Li J, Wu H, Li W, Yin L, Guo S, Xu X, et al. Downregulated miR-506 expression facilitates pancreatic cancer progression and chemoresistance via SPHK1/Akt/NF-κB signaling. Oncogene. 2016;35(42):5501–14. doi: 10.1038/onc.2016.90 27065335 PMC5078861

[43] Chen M-H, Yen C-C, Cheng C-T, Wu R-C, Huang S-C, Yu C-S, et al. Identification of SPHK1 as a therapeutic target and marker of poor prognosis in cholangiocarcinoma. Oncotarget. 2015;6(27):23594–608. doi: 10.18632/oncotarget.4335 26090720 PMC4695139

[44] Li W, Tian Z, Qin H, Li N, Zhou X, Li J, et al. High expression of sphingosine kinase 1 is associated with poor prognosis in nasopharyngeal carcinoma. Biochem Biophys Res Commun. 2015;460(2):341–7. doi: 10.1016/j.bbrc.2015.03.036 25778867

[45] Shi J, He Y, Sun J-X, Guo W-X, Li N, Xue J, et al. The impact of sphingosine kinase 1 on the prognosis of hepatocellular carcinoma patients with portal vein tumor thrombus. Ann Hepatol. 2015;14(2):198–206. doi: 10.1016/s1665-2681(19)30782-3 25671829

[46] Chang C, X U M, Wang J. Expression level of sphingosine kinase 1 and nuclear factor-κB p65 in non-small cell lung cancer and their relationship with tumor prognosis. Tianjin Medical Journal. 2014;:305–8.

[47] Yang L, Hu H, Deng Y, Bai Y. Role of SPHK1 regulates multi-drug resistance of small cell lung cancer and its clinical significance. Zhongguo Fei Ai Za Zhi. 2014;17(11):769–77. doi: 10.3779/j.issn.1009-3419.2014.11.01 25404266 PMC6000353

[48] Pan J, Tao Y-F, Zhou Z, Cao B-R, Wu S-Y, Zhang Y-L, et al. An novel role of sphingosine kinase-1 (SPHK1) in the invasion and metastasis of esophageal carcinoma. J Transl Med. 2011;9:157. doi: 10.1186/1479-5876-9-157 21936950 PMC3186754

[49] Zhuge Y, Tao H, Wang Y. Relationship between sphingosine kinase 1 expression and tumor invasion, metastasis and prognosis in gastric cancer. Zhonghua Yi Xue Za Zhi. 2011;91(39):2765–8. 22322056

[50] Liu G, Zheng H, Zhang Z, Wu Z, Xiong H, Li J, et al. Overexpression of sphingosine kinase 1 is associated with salivary gland carcinoma progression and might be a novel predictive marker for adjuvant therapy. BMC Cancer. 2010;10:495. doi: 10.1186/1471-2407-10-495 20846391 PMC2949806

[51] Watson C, Long JS, Orange C, Tannahill CL, Mallon E, McGlynn LM, et al. High expression of sphingosine 1-phosphate receptors, S1P1 and S1P3, sphingosine kinase 1, and extracellular signal-regulated kinase-1/2 is associated with development of tamoxifen resistance in estrogen receptor-positive breast cancer patients. Am J Pathol. 2010;177(5):2205–15. doi: 10.2353/ajpath.2010.100220 20889557 PMC2966780

[52] Li J, Guan H-Y, Gong L-Y, Song L-B, Zhang N, Wu J, et al. Clinical significance of sphingosine kinase-1 expression in human astrocytomas progression and overall patient survival. Clin Cancer Res. 2008;14(21):6996–7003. doi: 10.1158/1078-0432.CCR-08-0754 18980995

[53] Jiang T, Wu H, Lin M, Yin J, Tan L, Ruan Y, et al. B4GALNT1 promotes progression and metastasis in lung adenocarcinoma through JNK/c-Jun/Slug pathway. Carcinogenesis. 2021;42(4):621–30. doi: 10.1093/carcin/bgaa141 33367717

[54] Kim S. Bioinformatic analysis of sphingolipid metabolism-related genes for prognostic significance in patients with hepatocellular carcinoma using the cancer genome atlas liver cancer cohort. Quantitative Bio-Science. 2020;39(1):33–9.

[55] Qian H, Deng J, Lu C, Hou G, Zhang H, Zhang M, et al. Ceramide synthases: insights into the expression and prognosis of lung cancer. Exp Lung Res. 2021;47(1):37–53. doi: 10.1080/01902148.2020.1844345 33183094

[56] Wang J, Li R-Z, Wang W-J, Pan H-D, Xie C, Yau L-F, et al. CERS4 predicts positive anti-PD-1 response and promotes immunomodulation through Rhob-mediated suppression of CD8+Tim3+ exhausted T cells in non-small cell lung cancer. Pharmacol Res. 2023;194:106850. doi: 10.1016/j.phrs.2023.106850 37453674

[57] Hayama T, Hama K, Ozawa T, Fujiwara Y, Nozawa K, Matsuda K, et al. Ceramide synthase CERS4 gene downregulation is associated with KRAS mutation in colorectal cancer. Sci Rep. 2023;13(1):16249. doi: 10.1038/s41598-023-43557-1 37758931 PMC10533536

[58] Hartmann D, Lucks J, Fuchs S, Schiffmann S, Schreiber Y, Ferreirós N, et al. Long chain ceramides and very long chain ceramides have opposite effects on human breast and colon cancer cell growth. Int J Biochem Cell Biol. 2012;44(4):620–8. doi: 10.1016/j.biocel.2011.12.019 22230369

[59] Wofford W, Kim J, Kim D, Janneh AH, Lee HG, Atilgan FC, et al. Alterations of ceramide synthesis induce PD-L1 internalization and signaling to regulate tumor metastasis and immunotherapy response. Cell Rep. 2024;43(8):114532. doi: 10.1016/j.celrep.2024.114532 39046874 PMC11404065

[60] Newton J, Lima S, Maceyka M, Spiegel S. Revisiting the sphingolipid rheostat: Evolving concepts in cancer therapy. Exp Cell Res. 2015;333(2):195–200. doi: 10.1016/j.yexcr.2015.02.025 25770011 PMC4415605

