# Supplementary figures and images for: Sphingolipid metabolism-related genes B4GALNT1 and CERS4 as prognostic biomarkers in lung adenocarcinoma
Source: PLoS One. 2026 Feb 10;21(2):e0340437. doi: 10.1371/journal.pone.0340437 (PMC12890170; doi:10.1371/journal.pone.0340437)

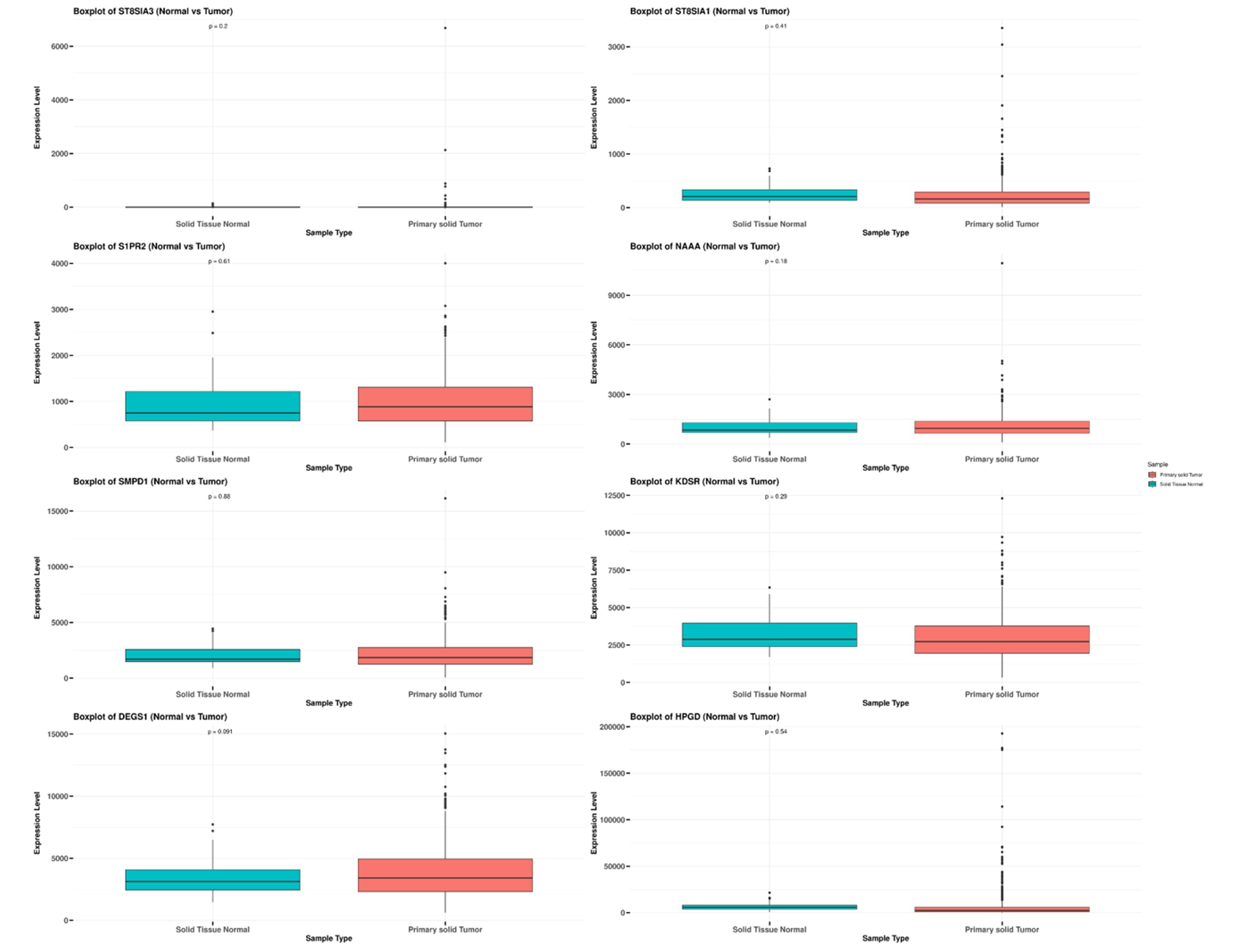

Supplement: S1 Fig — The graph shows the relative mRNA expression levels of sphingolipid metabolism-related genes, with eight genes showing no significant differences (P ≥ 0.05) when comparing LUAD tissues to NSTs. (JPEG) [file pone.0340437.s001.jpeg]

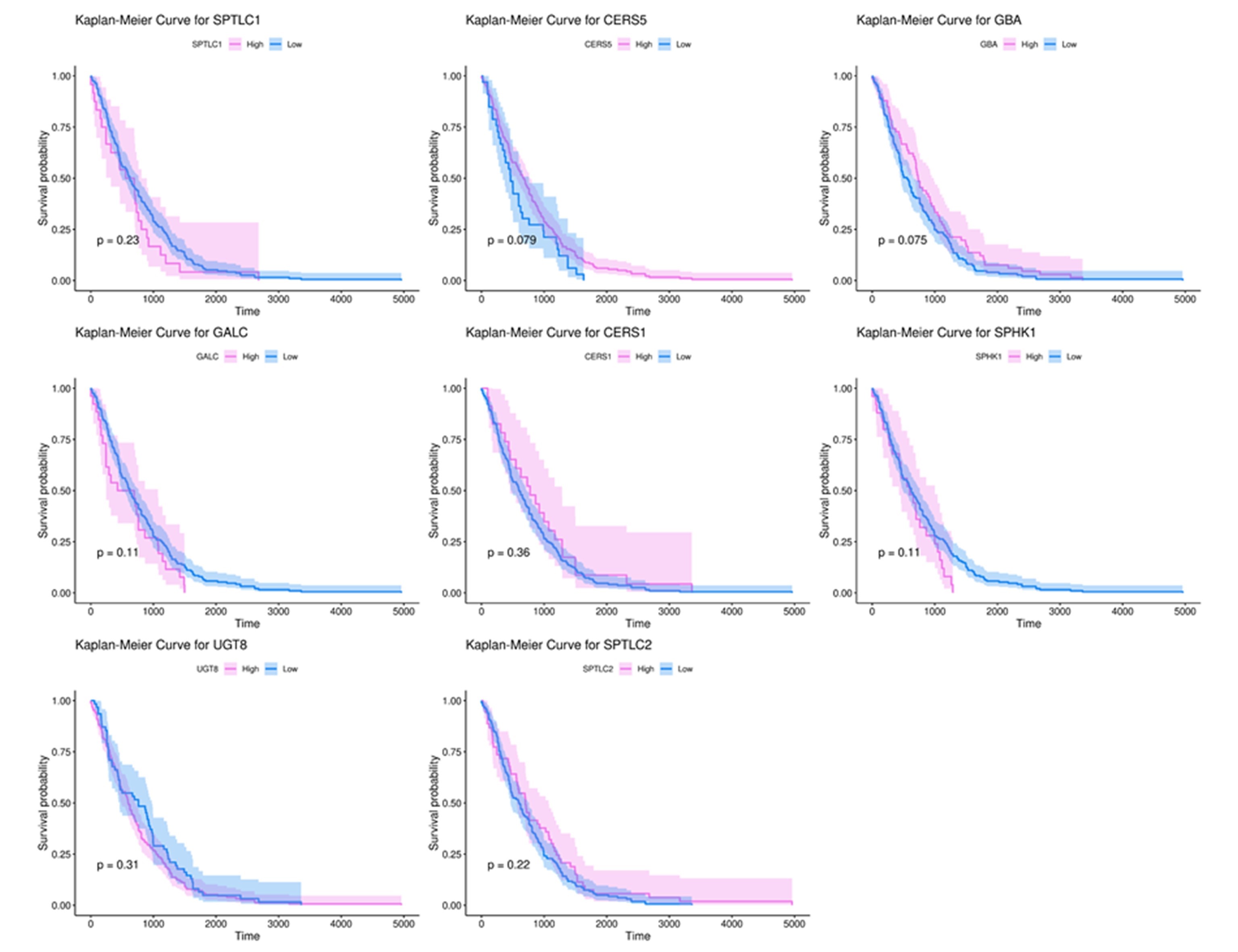

Supplement: S2 Fig — No significant correlations with overall survival were observed for the remaining eight sphingolipid metabolism-related genes, including SPTLC1, CERS5, GBA, GALC, CERS1, SPHK1, UGT8, and SPTLC2. (JPEG) [file pone.0340437.s002.jpeg]

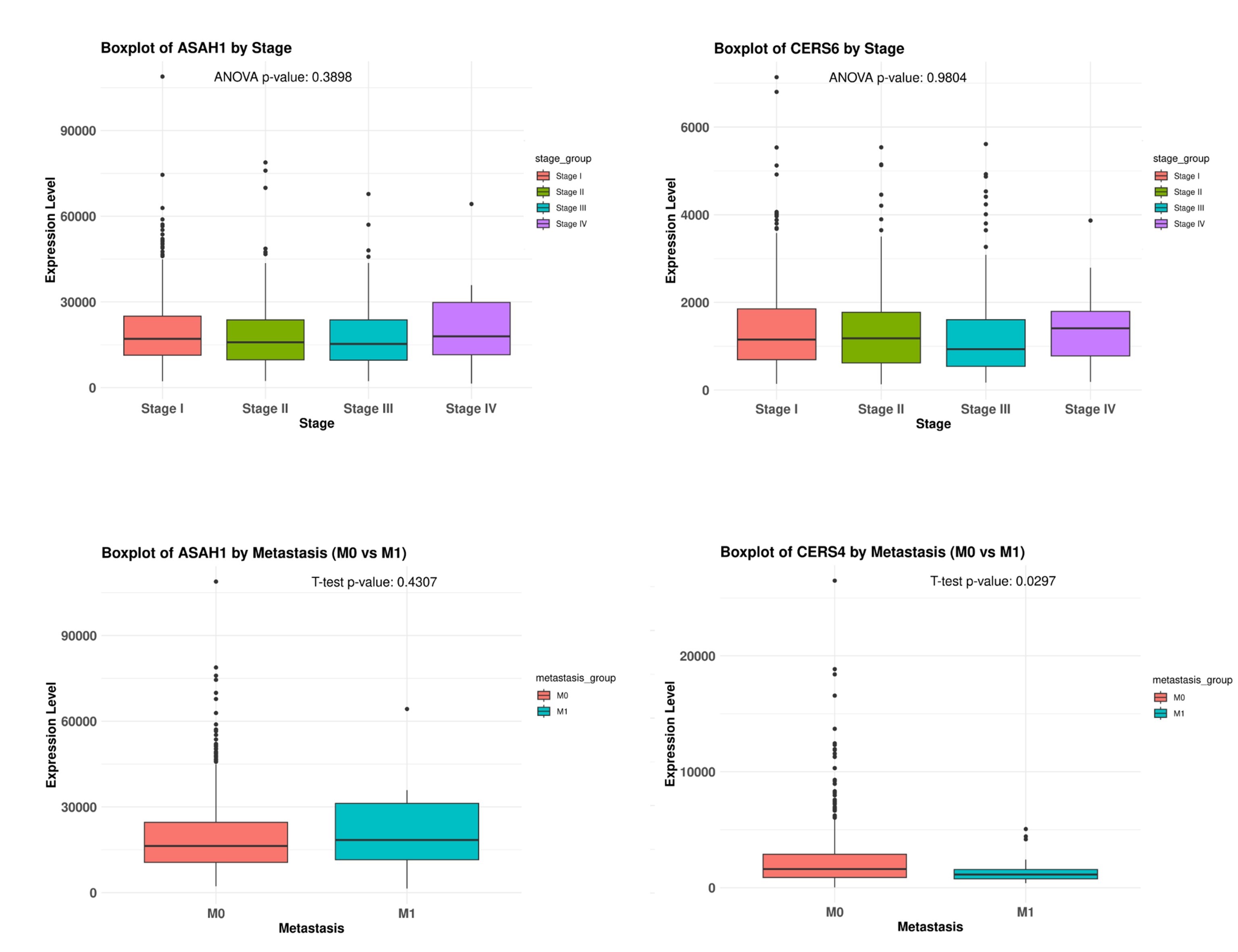

Supplement: S3 Fig — Box plots showing the mRNA expression levels of ASAH1 and CERS6 across different cancer stages (Stage I, II, III, and IV) and metastatic status (M0 vs. M1) in LUAD. (JPEG) [file pone.0340437.s003.jpeg]

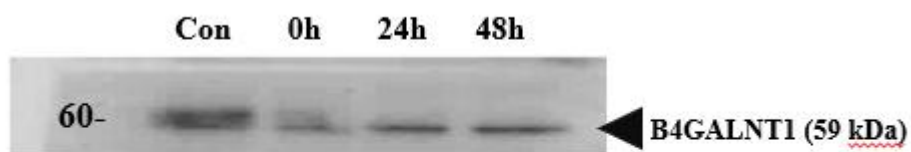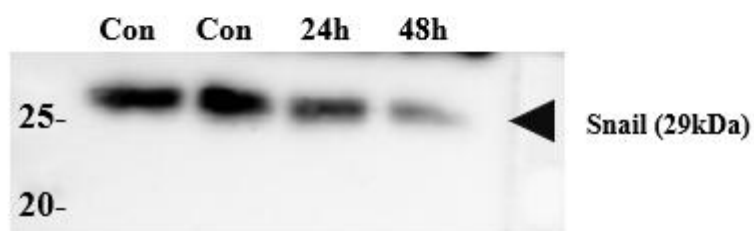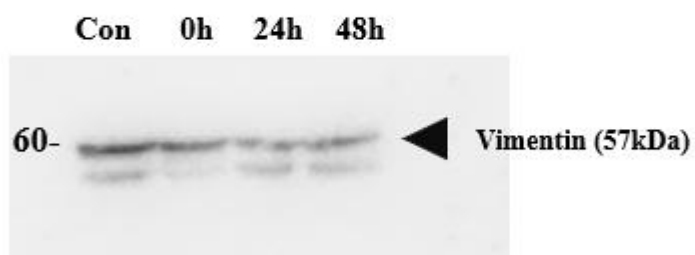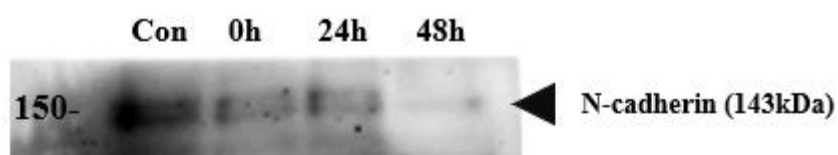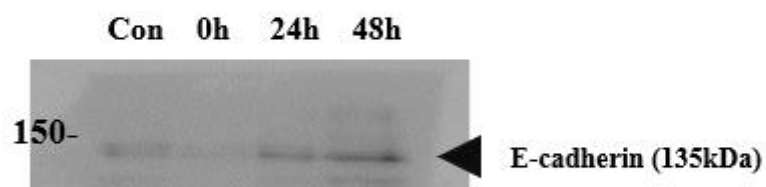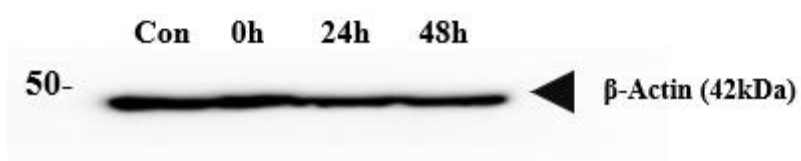

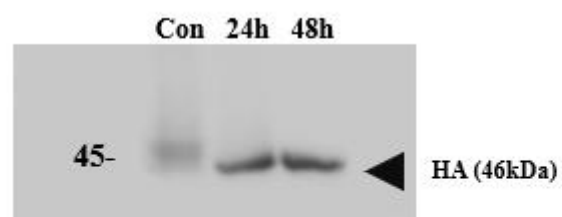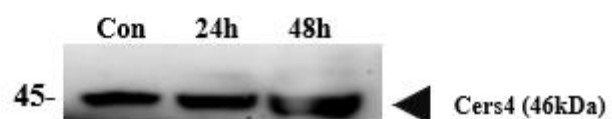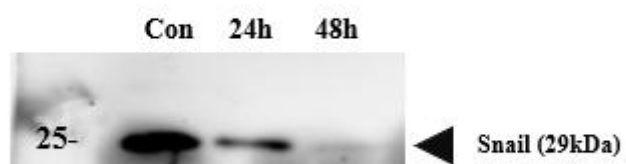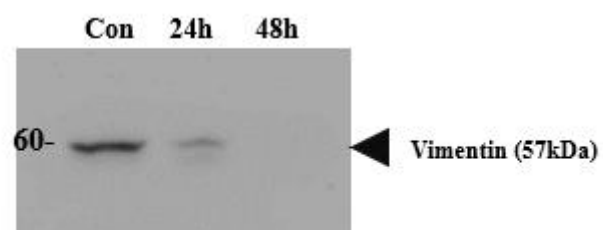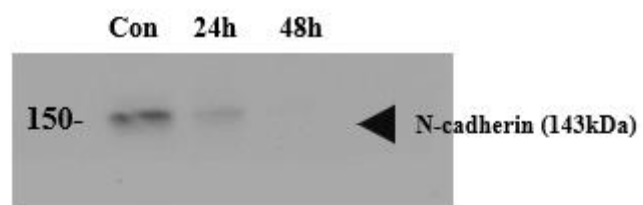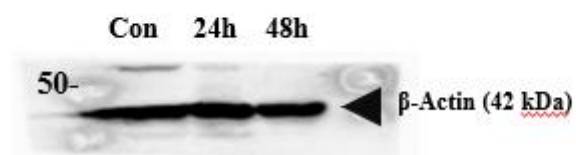

Supplement: S4 Fig — Related to Fig 6. (PDF) [file pone.0340437.s004.pdf]
