# Supplementary material for: Sphingolipid metabolism-related genes B4GALNT1 and CERS4 as prognostic biomarkers in lung adenocarcinoma
Source: PLoS One. 2026 Feb 10;21(2):e0340437. doi: 10.1371/journal.pone.0340437 (PMC12890170; doi:10.1371/journal.pone.0340437)
Supplement: S1 Table — (DOCX) [file pone.0340437.s005.docx]

S1 Table. Clinical information of LUAD patients from TCGA dataset.

| Clinical characteristics | Total (600) | % |
| --- | --- | --- |
| Age at diagnosis, y | 65.5 (33.4-88.9) |  |
| Futime, y | 2.11 (0-13.6) |  |
| Gender |  |  |
| Female | 325 | 54.2 |
| Male | 275 | 45.8 |
| Stage |  |  |
| I | 327 | 54.4 |
| II | 139 | 23.2 |
| III | 97 | 16.2 |
| IV | 28 | 4.7 |
| NA | 9 | 1.5 |
| Pathological T stage |  |  |
| T1 | 195 | 32.5 |
| T2 | 331 | 55.2 |
| T3 | 51 | 8.5 |
| T4 | 20 | 3.3 |
| TX | 3 | 0.5 |
| Pathological M stage |  |  |
| M0 | 407 | 67.8 |
| M1 | 27 | 4.5 |
| MX | 159 | 26.5 |
| NA | 7 | 1.2 |
| Pathological N stage |  |  |
| N0 | 381 | 63.5 |
| N1 | 110 | 18.3 |
| N2 | 87 | 14.5 |
| N3 | 2 | 0.3 |
| NX | 19 | 3.2 |
| NA | 1 | 0.2 |
| Vital status |  |  |
| Alive | 381 | 63.5 |
| Dead | 219 | 36.5 |
| Mutation status |  |  |
| EGFR | 47 | 7.8 |
| KRAS | 139 | 23.2 |
