# Supplementary material for: Sphingolipid metabolism-related genes B4GALNT1 and CERS4 as prognostic biomarkers in lung adenocarcinoma
Source: PLoS One. 2026 Feb 10;21(2):e0340437. doi: 10.1371/journal.pone.0340437 (PMC12890170; doi:10.1371/journal.pone.0340437)
Supplement: S2 Table — (DOCX) [file pone.0340437.s006.docx]

S2 Table. Clinical information of LUAD patients from GEO dataset.

| Clinical characteristics | Total (442) | % |
| --- | --- | --- |
| Age at diagnosis, y | 70 (38-89) |  |
| Futime, y | 2.26 (0.01-5.69) |  |
| Gender |  |  |
| Female | 240 | 54.3 |
| Male | 202 | 45.7 |
| Stage |  |  |
| I | 265 | 59.9 |
| II | 69 | 15.6 |
| III | 63 | 14.3 |
| IV | 17 | 3.8 |
| NA | 28 | 6.3 |
| Vital status |  |  |
| Alive | 298 | 67.4 |
| Dead | 122 | 27.6 |
| NA | 22 | 5.0 |
| Mutation status |  |  |
| EGFR | 47 | 10.6 |
| KRAS | 154 | 34.8 |
| TP53 | 111 | 25.1 |
